# Conditional generative adversarial network technology for OFDM system receiver signal detection

**DOI:** 10.1371/journal.pone.0334044

**Published:** 2025-10-14

**Authors:** Yang Liu, Peng Liu, Yu Shi, Xue Hao

**Affiliations:** Department of Communication Electronic Countermeasure, Aviation University of Air Force, Changchun, China; National Yang Ming Chiao Tung University, TAIWAN

## Abstract

In response to the limited detection accuracy of traditional orthogonal frequency division multiplexing systems in complex wireless channel environments, this study first uses conditional generative adversarial networks to construct a single input/output orthogonal frequency division multiplexing system signal detection model. On this basis, deep complex neural networks and quadratic concatenation of conditional information matrices are introduced to optimize the structure of conditional generative adversarial networks. Ultimately, a signal detection model for orthogonal frequency division multiplexing systems with multiple inputs and outputs is proposed. The experiment showed that the mean square error of channel detection for this new model could reach as low as 0.2. Compared to other advanced detection models, the channel equalization error of this new model was the lowest at 1.23%. In urban, suburban, and indoor environments, the channel equalization error of the research model was the lowest at 1.23%, the signal reception success rate was the highest at 98.72%, the detection accuracy was the highest at 96.45%, and the average detection time was the shortest at 11.62ms. The data demonstrate that the improved model exhibits significant advantages in signal detection precision and computational efficiency, especially in complex environments where it demonstrates higher robustness and adaptability. This provides a new solution for detecting orthogonal frequency division multiplexing signals in complex environments, with high application prospects.

## 1. Introduction

The growth of Wireless Communication (WLC) technology has driven the evolution of a novel generation of communication systems. Orthogonal Frequency Division Multiplexing (OFDM), as an efficient multi-carrier modulation technique, has become the core support of modern WLC. Due to its ability to effectively resist multipath fading, improve spectrum utilization, and reduce inter symbol interference, OFDM is widely used in fifth generation mobile communication, wireless local area networks, long-range evolution, and future terahertz communication systems [1,2]. However, despite the theoretical advantages of OFDM systems, their signal detection in practical applications still faces a series of challenges, such as Channel Estimation Errors (CEError), non-ideal synchronization, multipath interference, phase noise, and high peak to average ratio issues. These issues directly affect the receiver performance of OFDM systems, limiting their reliability and efficiency in complex wireless environments. Therefore, how to perfect the signal detection function of OFDM systems has become an essential research topic in the current field of WLC.

Bemani et al. proposed an embedded pilot assisted channel estimation scheme for OFDM by combining discrete reflection Fourier transform to enhance the signal detection effectiveness of OFDM in high mobility communication scenarios. This method significantly improved the performance of OFDM compared to the advanced multi-carrier schemes in high mobility scenarios [3]. Li J et al. proposed a composite multi-mode OFDM scheme with index modulation, which improves the spectral efficiency of OFDM by extending the index to the energy and constellation domains. It outperformed traditional OFDM related schemes, especially in high Signal-to-Noise Ratio (SNR) regions, verifying the precision of this new scheme for upper bound error detection [4]. Abed G A believed that while existing OFDM technology reduced signal interference, it still had a certain sensitivity to faults, leading to performance degradation. To this end, the scholar optimized OFDM technology by adding periodic prefixes and correcting carrier frequency offsets. The optimized OFDM significantly reduced the system’s Bit Error Ratio (BER) under various time offset conditions [5]. Xu X Y et al. proposed an OFDM technique based on discrete Hartley transform index modulation to enable the transmission of more index bits and improve its spectral efficiency. Compared with traditional methods, this new technology could significantly reduce the constellation order, thereby achieving better BER performance [6]. Rathod et al. integrated fast Fourier transform to reduce the reconfigurable peak in OFDM technology, improved the error vector amplitude through selective mapping, and proposed an optimization technique. This technology could improve the efficiency and complementary cumulative distribution function of OFDM communication process by reducing interference [7].

Generative Adversarial Network (GAN), as an advanced deep learning framework, has demonstrated superior performance in tasks such as data generation, feature learning, and denoising due to its adversarial training mechanism [8]. Compared to traditional deep learning methods, GAN can learn complex data distributions through game optimization of generators and discriminators, thereby improving the adaptability and generalization ability of the model. Mrabet et al. designed an auxiliary optimization method for OFDM transmission to effectively address deterministic nonlinear distortion and nonlinear effects between subcarriers in OFDM networks. The proposed method could indeed achieve better signal detection and transmission efficiency in real multi-line OFDM transmission [9]. Ayanoglu E et al. put forth a GAN-based OFDM systems to address spectrum sharing and mitigate security attacks by utilizing GAN algorithm for anomaly detection in signal classification. This method could improve the signal Detection Accuracy (DA) of the system through an autoencoder [10]. Mydhili S K found that there are non-stationary channel physical phenomena in estimating channel in OFDM. Given this, the scholar proposed a dual interactive GAN based on OFDM channel estimation using a hybrid Archimedean optimization and chimpanzee optimization algorithm optimization. This network achieved lower computational costs and lower error rates, with smaller Mean Square Errors (MSE) compared to other existing methods [11]. Hu et al. argued that traditional road-based automatic modulation classification methods cause confusion in OFDM-based signals due to differences in the available symbol lengths of OFDM systems. Therefore, researchers have proposed a novel signal modulation method combining GAN. This method exhibited higher performance, with an efficiency of 99.95% [12]. Alqahtani et al. Believed that although the least squares estimation method was cost-effective and widely used, there were still significant OFDM estimation errors. To this end, scholars have proposed a high margin error channel estimation method based on conditional self attention GAN. Compared with other existing methods, a lower BER has been achieved [13].

In summary, previous research has made significant progress in improving OFDM signal DA, reducing BERs, and optimizing channel estimation. However, most methods still face the problem of insufficient robustness in complex wireless environments, especially in multi-antenna and multi-channel conditions where performance is hard to satisfy the demands of efficient communication. Therefore, the paper constructs a Multi-Input/Output OFDM (MIMO-OFDM) signal detection model based on an improved Conditional GAN (CGAN). It effectively optimizes signal DA and computational efficiency by introducing Complex-Valued Neural Networks (CVNN) and Conditional Information Matrix Secondary Concatenation (CIMSC). The innovation lies in optimizing the GAN structure and enhancing the channel feature extraction capability, enabling the model to maintain high robustness and adaptability in complex channel environments. This study aims to provide a new and efficient solution for signal detection in OFDM systems with multiple antenna configurations and complex environments, and to offer theoretical support and practical basis for the optimization of future WLC systems.

## 2. Methods and materials

In response to the limited signal DA of traditional OFDM systems in complex wireless channel environments, this study first constructs a CGAN detection model suitable for Single Input/Output OFDM (SISO-OFDM) to optimize the effect of CEErrors on signal recovery. Subsequently, in this system, CVNN is introduced to enhance the ability of complex signal feature extraction, and combined with CIMSC strategy to optimize the utilization of channel information, improving the adaptability and stability of signal detection. Finally, this study proposes a MIMO-OFDM model based on improved CGAN.

### 2.1. Construction of SISO-OFDM system based on CGAN

**Fig 1:**
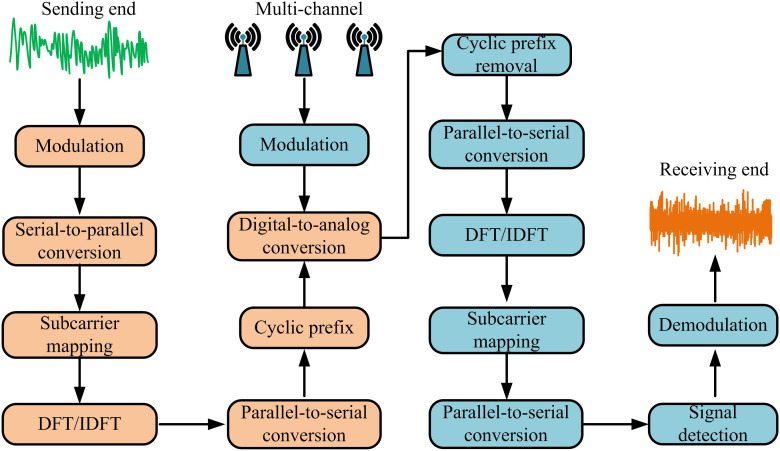
The system principle of OFDM.

OFDM is a multi-carrier modulation technique, widely utilized in modern WLC systems [14]. The core idea is to split high-speed data streams into multiple low-speed parallel subcarriers for transmission, aiming to improve spectrum utilization and enhance resistance to multipath fading [15]. The key advantage of OFDM system lies in its excellent resistance to inter symbol interference, which can offset the impact of channel delay extension through cyclic prefix, thereby improving the robustness of the system. The system principle of OFDM is shown in Fig 1 [16,17].

In Fig 1, the OFDM system mainly consists of three parts: the transmitter, the channel, and the receiver. The transmitting end includes modulation module, serial parallel conversion, subcarrier mapping, Discrete Fourier Transform (DFT), and Inverse DFT (IDFT), parallel to serial conversion, cyclic prefix addition, and digital to analog conversion module. The channel section mainly considers the impact of multipath channels on signals. The receiving end includes analog-to-digital conversion, cyclic prefix removal, serial parallel conversion, DFT/IDFT conversion, channel equalization, parallel to serial conversion, and demodulation modules [18,19]. The specific process is that the input bit stream is first modulated in the modulation module, and then converted to allocate the data to multiple subcarriers. After subcarrier mapping, the DFT/IDFT module is input for frequency domain to time domain conversion, and then converted to form a single time domain signal. The cyclic prefix is added to combat multipath interference and ultimately converted before being transmitted to the wireless channel. At the receiving end, the signal is first converted back to a digital signal, then the cyclic prefix is removed and converted back to the frequency domain in the DFT/IDFT module. After compensating for channel fading and noise effects through channel equalization, the original data are converted and demodulated [20,21]. In the SISO-OFDM system, it is assumed that the symbol sequence transmitted by the transmitting end is $X=\{X_{0},X_{1},\ldots ,X_{N-1}\}$ , where N represents the number of subcarriers. Each symbol is converted to the time domain through IDFT to form a transmission signal, and the formula is given by equation (1) [22].


$$x_{n}=\frac{\text{1}}{\sqrt{n}}\sum_{k=0}^{N-1}{X_{k}}^{e^{j\frac{2\pi kn}{N}}},n=0,1,\cdots ,N-1$$ (1)

In equation (1), $x_{n}$ is the signal value of the n -th sampling point, which is the signal sent in the time domain. $X_{k}$ denotes the modulation symbol on the k -th subcarrier at the transmitting end. N is the gross of subcarriers in the OFDM system. j is an imaginary unit. $\backslash {(}^{e^{j\frac{2\pi kn}{N}}}$ is the IDFT transformation coefficient. In wireless channels, signals propagate through multipath, assuming that the impulse response of the channel is a finite impulse response model, as given by equation (2).


$$h(t)=\sum_{l=0}^{L-1}h_{l}\cdot \delta (t-\eta_{l})$$ (2)

In equation (2), h(t) is the impulse response of the wireless channel. $h_{l}$ is the fading coefficient on the l -th path. δ(t−η) is the unit impulse function. $\eta_{l}$ is the propagation delay of the l -th path. L is the number of multipath paths in the channel. After adding Gaussian white noise, the received signal can be represented as shown in equation (3).


$$y_{n}=\sum_{l=0}^{L-1}hl\cdot x_{n-\eta_{l}}+\omega_{n}$$ (3)

In equation (3), $y_{n}$ is the received value at the n -th sampling point, which is the signal at the receiving end in the time domain. $x_{n-\eta_{l}}$ is the transmitted signal at the n -th time point after multipath propagation. $\omega_{n}$ is additive Gaussian white noise. At the receiving end, DFT is performed on $y_{n}$ to gain the received symbol, as given by equation (4) [23].


$$\begin{array}{cc} {}*20cY_{k}=H_{k}X_{k}+W_{k}, & k=0,1,\cdots ,N-1 \end{array}$$ (4)

**Fig 2:**
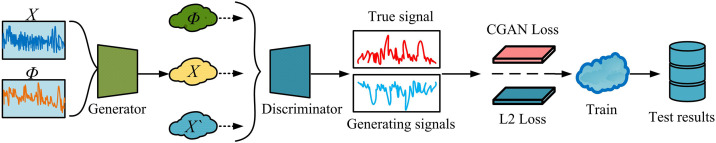
The structure of CGAN.

$Y_{k}$ is the k -th subcarrier signal of the receiving end in the frequency domain. $H_{k}$ means the channel frequency response on the k -th subcarrier. $X_{k}$ denotes the modulation symbol of the transmitting end on the k -th subcarrier. $W_{k}$ is the Gaussian white noise after DFT transformation. Channel equalization usually uses minimum MSE or maximum likelihood estimation methods, but these methods have limited detection performance in complex wireless environments due to CEErrors, making it difficult to adapt to dynamic channel changes [24,25]. Therefore, this study introduces CGAN. Compared with other methods, CGAN can not only maintain good detection performance even with large CEErrors but also adaptively learn statistical characteristics in different channel environments, effectively responding to dynamic channel changes [26]. The CGAN structure in OFDM system is shown in Fig 2.

**Fig 3:**
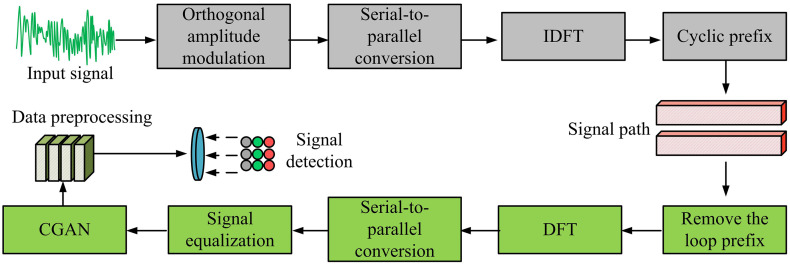
Signal detection model of OFDM receiver based on CGAN.

The structure in Fig 2 mainly consists of a generator and a discriminator, using adversarial training mechanism to optimize signal detection performance. In the offline training phase, the generator receives input signal X and conditional information, such as channel state information Φ. By learning channel characteristics, it generates a signal G(X,Φ) that is close to the true distribution and passes it to the discriminator for evaluation. The generator consists of three complex convolutional layers and two fully connected layers. Each convolutional layer uses a 3 × 3 convolution kernel and 64 filters, with the activation function being ReLU. The fully connected layers use tanh to enhance nonlinear expression capabilities; The discriminator consists of two convolutional layers and two fully connected layers. Each convolutional layer also uses a 3 × 3 convolutional kernel and 128 filters, with the convolutional layer activation function being Leaky ReLU. The final fully connected layer uses Sigmoid as the activation function to output the true/false classification probability. The discriminator calculates the discrepancies between the generated and real signals through the discriminant function D(X,Φ), and continuously optimizes the generator through adversarial training to make its output closer to the ideal detection result. The goal of the discriminator is to distinguish between the real signal D(G(X,Φ),Φ)=0 and the generated signal D(X,Φ)=1, and to jointly optimize the generator through CGAN Loss and L2 Loss, so that it can generate high-quality signal detection results under different channel conditions. In the online application stage, the trained generator can be directly used for signal detection in OFDM systems without relying on discriminators, thereby reducing computational overhead and improving real-time detection performance. At this point, combined with CGAN, this study proposes a receiver signal detection model for SISO-OFDM systems, as shown in Fig 3.

In Fig 3, the SISO-OFDM model based on CGAN includes a transmitter, a channel, and a receiver. The sender first performs orthogonal amplitude modulation on the input bit stream, converts it from serial to parallel, and then transforms it into the time domain through IDFT. It adds a cyclic prefix to enhance its ability to resist multi-path interference and transmits it through a wireless channel. The channel is influenced by multi-path effects and additive noise, leading to the distortion in the received signal. The receiving end first performs prefix removal and IDFT transformation to recover the frequency domain signal, and then uses traditional equalization methods to preliminarily recover the signal. The CGAN module further optimizes the signal detection process, where the generator receives the balanced signal and generates an optimized signal. The discriminator continuously optimizes the generator through adversarial training to make its output closer to the original transmission signal. Finally, after signal detection and data recovery, high-precision received signals are obtained. Among them, the CGAN input signal is constructed as shown in equation (5).


$${\hat{X}}_{k}^{CGAN}=G(Y_{k},{\hat{H}}_{k},Z)$$ (5)

In equation (5), ${\hat{X}}_{k}^{CGAN}$ is the estimated value of the optimized signal generated by CGAN. G is a generator network. Z is a random noise vector. The discriminator is used to distinguish between generated signals and real signals, and its training process is shown in equation (6).


$$D(X_{k},H_{k})=P(X_{k}|X_{k},{\hat{H}}_{k})$$ (6)

In equation (6), D is the discriminator network. P is the discriminator’s estimated probability of whether the input data is true or not. To enhance the accuracy of the signal, this study adds MSE constraints to its loss function. The constraints and overall objective function are shown in equation (7).


$$\{\begin{array}{c} {}*20cL_{MSE}=E\left[{\left\|X_{k}-G(Y_{k},{\hat{H}}_{k},Z)\right\|}^{2}\right]\\ L_{total}=L_{GAN}+\lambda L_{MSE} \end{array}$$ (7)

In equation (7), E is the expected value. $L_{GAN}$ is to combat losses. $L_{MSE}$ is the MSE loss. λ is the weight parameter of the loss function.

### 2.2. Construction of MIMO-OFDM model based on improved CGAN

**Fig 4:**
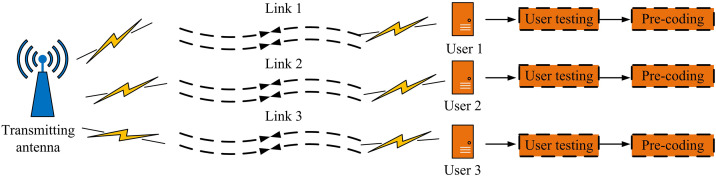
MIMO-OFDM signal transmission system.

After constructing the SISO-OFDM system, it is found that CGAN can learn channel characteristics through a generator and optimize signal detection strategies using a discriminator, improving the DA and anti-interference ability of the system. However, OFDM has been extensively utilized in MIMO architectures to enhance system capacity, spectral efficiency, and anti-interference performance [27,28]. In a MIMO environment, due to multiple antennas simultaneously transmitting signals, the receiving end not only needs to perform channel equalization on the signals but also needs to solve the problems of inter channel interference and spatial correlation, making the signal detection task more complex. The signal transmission of MIMO-OFDM system is shown in Fig 4 [29,30].

**Fig 5:**
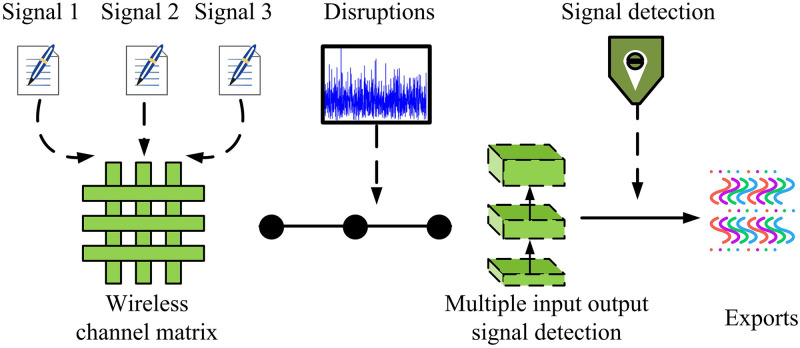
Signal detection diagram of MIMO-OFDM.

In Fig 4, the MIMO-OFDM system adopts a multi-antenna transmission mode, which simultaneously transmits data to multiple receiving ends through multiple transmitting antennas, improving spectrum utilization and transmission efficiency. In the figure, the uplink transmission of MIMO-OFDM system involves multiple user devices simultaneously sending signals to the base station. The base station receives data streams from multiple users and recovers the original information through multi-user detection and signal separation techniques. Through downlink transmission, the base station uses multiple antennas to send data to multiple user devices, optimizes signal transmission through precoding and beamforming techniques, reduces interference between users, and improves signal quality. In MIMO mode, the transmission of signals between multiple antennas makes the signal detection process at the receiving end more complex than SISO [31]. Due to multiple antennas simultaneously transmitting and receiving data, the receiving end not only needs to deal with conventional channel fading and noise interference but also needs to deal with inter-channel interference and inter-antenna interference caused by multiple transmission streams. The signal detection of MIMO-OFDM is displayed in Fig 5.

**Fig 6:**
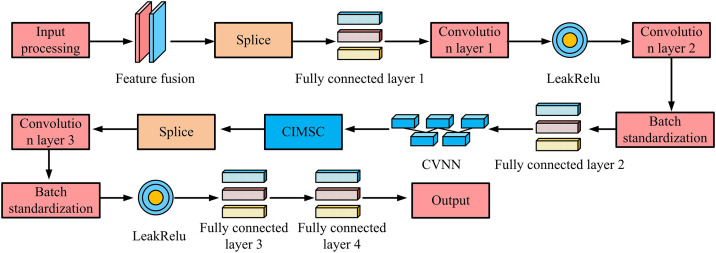
CVNN-CIMSC-CGAN process.

In Fig 5, the process mainly includes four parts: signal input, channel transmission, noise interference, and signal detection. Firstly, multiple transmitting antennas transmit data simultaneously. The input signal propagates through wireless channel matrix H. The multipath effect and fading characteristics of the channel can have an impact on the signal. Due to the use of multiple receiving antennas in MIMO-OFDM systems, the receiving end will simultaneously receive multiple data streams. Moreover, due to cross interference between channels, the signal at the receiving end will be affected by channel noise and inter antenna interference, resulting in the superposition of interference term n on the received signal. At the receiving end, the detection module needs to restore the detection signal $\hat{x}$ that is closest to the original input and process the received signal. To further improve the DA and computational efficiency of OFDM systems in complex channel environments, this study attempts to optimize the CGAN structure and introduce CVNN and CIMSC strategies to enhance the model’s ability to learn MIMO channel characteristics. Compared to traditional real valued neural networks, CVNN can directly process complex signals, avoiding conversion losses between the complex and real domains, enabling the neural network to learn the amplitude and phase characteristics of MIMO channels more accurately. In addition, CIMSC introduces additional channel state information at the input of CGAN generator and discriminator, and performs secondary concatenation during training to enable the model to more fully utilize channel correlation. The optimized CGAN process is shown in Fig 6.

In Fig 6, the process includes six steps: input processing, feature fusion, deep complex convolution, concatenation optimization, fully connected processing, and final signal generation. In terms of parameters, in the improved multiple-input-output CGAN model, the generator first splices the inputs of the received signal and channel state matrix, after four complex convolutional layers and two complex fully connected layers, the convolution kernel size is set to 3 × 3, the first two convolutional layers are activated by ReLU, and the last two convolutional layers use Leaky ReLU to avoid gradient vanishing, and the output layer adopts the tanh function to generate the detection signal; the discriminator includes three complex convolutional layers and two fully connected layers, the convolutional kernel is also 3 × 3, the convolutional layers all use Leaky ReLU, and the final layer outputs the true-false discrimination result through the Sigmoid function. Firstly, the input data include the channel estimation matrix $H_{ls}$ and the received signal y. The two are concatenated in the initial stage to enhance the utilization of channel information. Subsequently, the data are processed through CVNN, utilizing the computational advantages of the complex domain to enhance the ability to learn amplitude and phase information of OFDM signals. After feature extraction, CIMSC is performed to enhance the generator’s ability to capture channel correlation. Finally, the data are processed through Fully Connected Layers (FCLs) and activation functions to generate optimized detection signals. The calculation of initial input concatenation is shown in equation (8).


$$X_{cimsc}^{\left(1\right)}=Concat\left(H_{ls},y,\;H_{ls}^{H}y\right)$$ (8)

In equation (8), $X_{cimsc}^{\left(1\right)}$ is the initial concatenated input matrix. $H_{ls}$ is the channel information estimated by the least squares method. y is the received signal. $H_{ls}^{H}y$ is the product of the complex conjugate channel matrix and the received signal. The complex signal feature extraction formula of CVNN is shown in equation (9).


$$F_{cvnn}=\sigma \left(W_{r}*X_{cimsc}^{\left(1\right)}+jW_{i}*X_{cimsc}^{\left(1\right)}+b_{c}\right)$$ (9)

In equation (9), $F_{cvnn}$ is the feature matrix extracted by the complex convolutional network. $W_{r}$ and $W_{i}$ are the real and imaginary parts of complex convolution kernels. $b_{c}$ is the bias term. σ is a nonlinear activation function. The expression of channel information enhancement through secondary splicing is shown in equation (10).


$$X_{cimsc}^{\left(2\right)}=Concat\;F_{cvnn},H_{ls},diag(H_{ls}\cdot H_{ls}^{H})$$ (10)

In equation (10), $X_{cimsc}^{\left(2\right)}$ is the optimized input matrix. $diag(H_{ls}\cdot H_{ls}^{H})$ is the autocorrelation feature of the channel matrix. The quadratic cascade of CIMSC can be formalized as a two-stage mapping, where the least squares estimated channel matrix is firstly spliced with the received signal to obtain the input matrix in the initial splicing stage, and subsequently the channel autocorrelation matrix is spliced again after complex convolution of the extracted features, thus realizing double constraints on the feature level and channel level, whose mathematical form can be denoted as $X^{\prime }=[f(W_{c}*h+r),H^{H}H]$, where f(·) denotes the nonlinear mapping after the complex convolution, $W_{c}$ is the convolution kernel, h is the received signal, r is the bias term, and $H^{H}H$ is the channel autocorrelation matrix. Unlike relying only on a single splicing when CIMSC is not employed, the secondary cascade is able to make more full use of the channel correlation during the training process, thus reducing the detection bias and improving the convergence stability of the generator. Previous experiments have shown that, without using CIMSC, the mean square error of the model typically remains between 0.35 and 0.4, while using CIMSC can reduce it to below 0.25; In low signal-to-noise ratio scenarios, the bit error rate (BER) typically remains at the 10^−3^ level without CIMSC, but can be further reduced to 10^−4^ or even 10^−5^ with CIMSC, indicating that the secondary cascade not only improves channel information utilization but also effectively enhances the generator’s convergence stability and generalization capability. At this point, the optimized channel feature input generator is mapped to a high-dimensional feature space through a complex FCL, as shown in equation (11).


$$F_{fc}=\sigma \left(W_{f}X_{cimsc}^{\left(2\right)}+b_{f}\right)+j\sigma \left(W_{f}^{\prime }X_{cimsc}^{\left(2\right)}+b_{f}^{\prime }\right)$$ (11)

In equation (11), $F_{fc}$ is the output feature of the complex FCL. $W_{f}^{\prime }$ and $W_{f}$ are the imaginary and real parts of the weight matrix of the FCL. $b_{f}$ and $b_{f}^{\prime }$ are bias terms. The generator outputs optimized detection signals for adversarial training with real signals, as shown in equation (12).


$$\begin{array}{c} \hat{X}=G(F_{fc})=\mathrm{\backslash arg}\min{\mathrm{\backslash nolimits}}_{X}{\left\|X-G(F_{fc})\right\|}^{2}+\vartheta {\left\|H_{ls}G(F_{fc})-y\right\|}^{2} \end{array}$$ (12)

**Fig 7:**
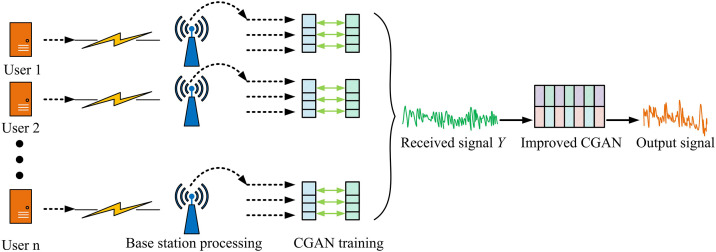
MIMO-OFDM model.

In equation (12), $\hat{X}$ is the optimized signal estimation value. ϑ is the regularization parameter. A MIMO-OFDM model in complex environments is proposed based on the SISO-OFDM model, as shown in Fig 7.

In Fig 7, the model has four core components: multi-user signal input, MIMO channel transmission, base station reception and processing, and GAN training network. Firstly, multiple user devices simultaneously send signals to the base station. The signal propagates through MIMO channels and is affected by multipath effects, channel fading, and interference noise. The base station receiver is composed of multiple antenna units. Each receiving port receives signals propagated through different paths and synthesizes them into the received signal Y. By improving the GAN generator to learn channel characteristics, signal detection results that are closer to the true distribution are generated, and the detection strategy is optimized through a discriminator to improve the accuracy and robustness of signal recovery. Ultimately, by improving the CGAN training and optimizing the detection signal $\hat{X}$ as the output, the OFDM system can still maintain excellent signal detection performance in complex channel environments.

### 2.3. Proposed method explanation

Conditional Generative Adversarial Network (CGAN) introduces conditional constraint information on the basis of traditional GAN, which is able to incorporate channel state information in the inputs of both generator and discriminator, so that the generator learns a signal representation that is more in line with the characteristics of the wireless channel during the adversarial training process. The basic idea is to make the generator output not only close to the real signal distribution, but also maintain the stability and robustness of signal detection under the conditional information constraints by minimizing the weighted sum of the adversarial loss and the mean-square error loss between the generator and the discriminator. In the proposed OFDM signal detection model, CGAN can effectively mitigate the detection bias caused by channel estimation error and realize adaptive signal recovery under complex channel conditions.

Deep Complex-Valued Neural Networks (CVNN), on the other hand, extends the traditional real-valued neural networks to the complex domain so that the model can directly handle complex inputs. The advantage is that it avoids the split conversion from complex to real, reduces the loss of phase information, and is able to more accurately model the amplitude and phase characteristics of OFDM signals through complex convolution and complex fully connected layers. In the proposed improved CGAN structure, CVNN is used to enhance the feature extraction capability of the generator and the discriminator, so that the model can better capture the complex correlation of the channel in a multi-input-output environment, thus improving the accuracy and stability of signal detection.

## 3. Results

To verify the practical application effect of the proposed model, a suitable experimental environment was first set up. Two types of publicly available datasets were used to conduct hyperparameter selection testing, ablation testing, and performance comparison testing with advanced models to verify the detection performance of the research model. In addition, channel capacity and Signal Reception Rate (SRR) tests were conducted in three types of environments. Its superior performance in various environments was further verified by using Frequency Offset Rate (FOR), Symbol Error Rate (SER), and Channel Equalization Error (CEE) as indicators, especially maintaining good DA and robustness in complex environments.

### 3.1. Performance testing of MIMO-OFDM model

This study uses the Wireless InSite MIMO Channel Dataset (WIMC-DS) and DeepMIMO Dataset (DeepMIMO-DS) as test data sources. Among them, WIMC-DS is generated based on the Wireless InSite radio propagation simulation tool, containing MIMO-OFDM channel data in different environments, including urban, suburban, and indoor scenes. The data cover channel state information, path loss, delay propagation, multipath fading, and other information under 2 × 2, 4 × 4, 8 × 8, and 16 × 16 MIMO configurations. DeepMIMO-DS is generated based on a real ray tracing simulation environment, covering different multi-channel configurations and channel conditions, providing a complete channel state information matrix, channel response, noise interference data, and supporting multiple channel modeling methods. In the training configuration, all the networks use Adam optimizer, the learning rate of generator and discriminator is tuned in the range of 0.0001 ~ 0.0002, the batch size is 64, the number of training rounds is 200, the loss function combines the adversarial loss with MSE constraints, the regularization parameter is taken as 0.001, and the earliest stopping strategy is used in the training in order to avoid overfitting. Table 1 lists the specific experimental parameters.

**Table 1:** Experimental configuration and parameters.

Configuration	Parameter
Operating system	Windows 11 Professional 64-bit
CPU	Intel i9-12900K 3.2GHz
GPU	NVIDIA RTX 3090 24GB of video memory
Memory	32GB
Python language environment	Python 3.9.12
Deep learning framework	PyTorch 1.12.1
Hard disk	1TB SSD
MIMO antenna configuration	2 × 2, 4 × 4, 8 × 8, 16 × 16
Number of OFDM subcarriers	128, 256, 512, 1024
Cyclic Prefix length	1/4, 1/8, 1/16 of symbol length
Sampling rate	20 MHz
SNR range	0-30 dB
Training dataset size	100000 samples
Testing dataset size	20000 samples
Batch size	64
Number of epochs	200

**Fig 8:**
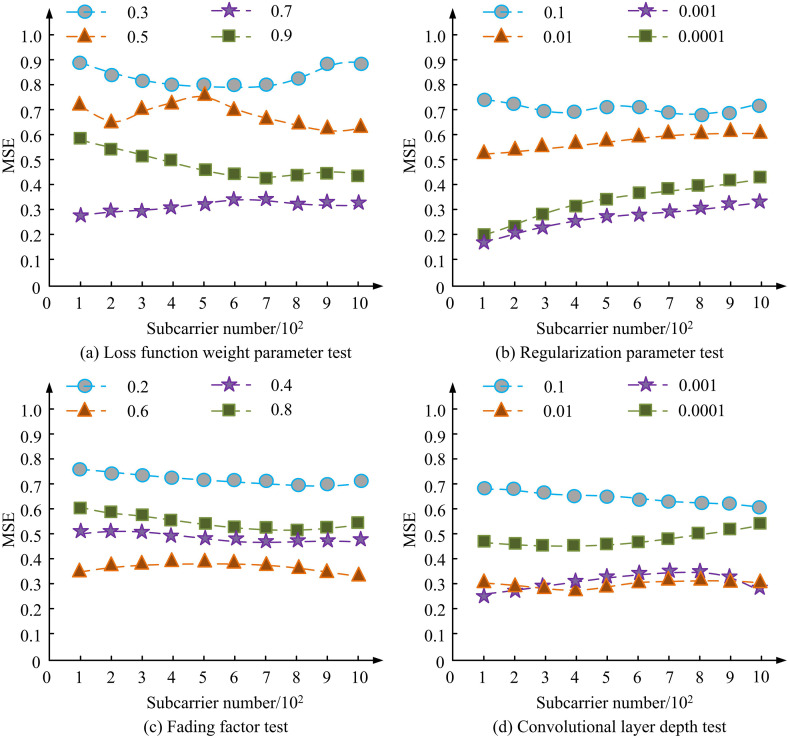
Hyperparameter selection test result.

Based on Table 1, this study first conducts value selection tests on the four types of hyperparameters that have the greatest impact on model performance, namely the loss function weight parameter λ, the regularization parameter ϑ and Decay coefficients and convolutional layer depth. The test results based on the MSE of channel detection are shown in Fig 8.

**Fig 9:**
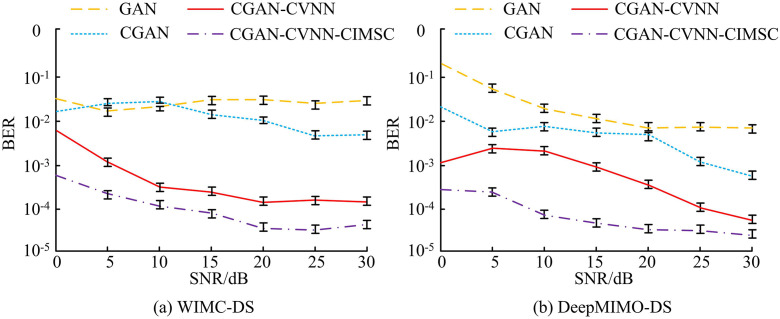
Ablation test results.

Fig 8a shows the test results of selecting the value of the loss function weight parameter, Fig 8b shows the test results of selecting the value of the regularization parameter, Fig 8c shows the test results of selecting the value of the decay coefficients, and Fig 8d shows the test results of selecting the value of the depth of the convolutional layer. From Fig 8a, it can be seen that the model performance is optimal when the weight parameter of the loss function takes the value of 0.7, and the MSE stabilizes at about 0.3, indicating that an optimal balance between the adversarial loss and the mean squared error is reached at this time; whereas, when the weight parameter is too small, such as 0.3, the MSE is close to 0.9, and the detection performance decreases significantly, indicating that the over-reliance on the MSE weakens the antagonistic learning ability of the generator; and when the weight parameter is too large, such as 0.9, the MSE also rises to around 0.5, indicating that over-reliance on adversarial loss leads to model underfitting. From Fig 8b, the regularization parameter at 0.001 has the lowest MSE of about 0.25, indicating that overfitting can be effectively suppressed at this time without affecting the signal feature learning, and when the parameter is increased to 0.01 the MSE rises to 0.6, and when it is further increased to 0.1 the MSE is more than 0.7, showing that overly strong regularization constraints will limit the network expressive ability. From Fig 8c, the fading coefficient in the interval of 0.4 to 0.6 maintains the MSE within 0.35 for optimal performance, while at 0.2 the MSE maintains around 0.7, indicating that too small fading coefficients are unable to effectively capture the multipath channel features; at 0.8 the MSE is close to 0.5, and the performance is also degraded, suggesting that too large fading coefficients introduce unwanted noise amplification. From Fig 8d, it can be seen that the depth of the convolutional layer is about 0.3 when the MSE is the lowest in three layers, reflecting that the deep structure can effectively extract complex channel features; when the depth is only one layer, the MSE is as high as 0.6, which indicates that the shallow network is not enough to extract the ability; when the depth is increased to four layers, the MSE is close to that of the three layers, but it is slightly inferior to that in the stability of the training and the computational overhead is obviously increased, so the three-layer convolution is the best balance between the precision and the efficiency. Therefore, three-layer convolution achieves the best balance between accuracy and efficiency. This study continues with ablation testing using BER as the indicator, as shown in Fig 9.

Fig 9a and 9b show the results from the WIMC-DS and DeepMIMO-DS datasets. In Fig 9a, when the SNR is 0dB, the BER of the GAN model is 10^−1^, while the BER of CGAN, CGAN-CVNN, and CGAN-CVNN-CIMSC are 10^−2^, 10^−3^, and 10^−4^. As the SNR grows to 5dB, the BER of CGAN-CVNN and CGAN-CVNN-CIMSC further decreases, but the BER of CGAN-CVNN-CIMSC is always better than other models, with the lowest BER being 10^−5^. In Fig 9b, at SNR0dB, the BER of GAN is 10^−1^, while the BER of other models is 10^−2^, 10^−2^, and 10^−3^. As the SNR increases, CGAN-CVNN and CGAN-CVNN-CIMSC continue to perform well, with BER decreasing to 10^−3^ and 10^−4^ at SNR of 20dB. At an SNR of 30dB, the BER of CGAN-CVNN-CIMSC is close to 10^−5^, which is significantly better than other models. Overall, CGAN-CVNN and CGAN-CVNN-CIMSC perform better than GAN at various SNRs, especially at low SNRs, where CGAN-CVNN-CIMSC exhibits optimal performance. This study introduces similar signal detection models, namely Deep GAN (DGAN), Self-Attention-based GAN (SAGAN), and Variational Autoencoder-based Signal Detection Model (VAE-SDM). A comparison is made using CEE, Signal Reception Success Rate (SRSR), DA, and Average Detection Time (ADT) as indicators, as listed in Table 2.

**Table 2:** Index test results of different detection models.

Data set	Model	CEE/%	SRSR/%	DA/%	ADT/ms
WIMC-DS	DGAN	2.38	97.65	94.12	14.3
	SAGAN	3.45	95.23	92.67	16.48
	VAE-SDM	4.12	93.89	91.34	18.67
	Our model	1.23	98.72	96.45	12.05
DeepMIMO-DS	DGAN	3.58	96.12	93.45	15.12
	SAGAN	4.25	94.38	91.89	17.01
	VAE-SDM	5.09	92.45	90.23	19.38
	Our model	1.13	99.05	97.25	11.62

In Table 2, on the WIMC-DS dataset, the CEE of the research model is 1.23%, lower than other models, while the SRSR and DA are 98.72% and 96.45%, and higher than DGAN, SAGAN, and VAE-SDM. On the DeepMIMO-DS dataset, the proposed model also performs well, with CEE of 1.13%, SRSR and DA of 99.05% and 97.25%. In addition, the ADT of the research model is also the shortest, at 12.05ms and 11.62ms, demonstrating its advantage in computational efficiency. Overall, the proposed model outperforms other compared signal detection models in various indicators, especially in channel equalization and DA, demonstrating its higher robustness and efficiency in complex environments.

### 3.2. Simulation testing of MIMO-OFDM model

**Fig 10:**
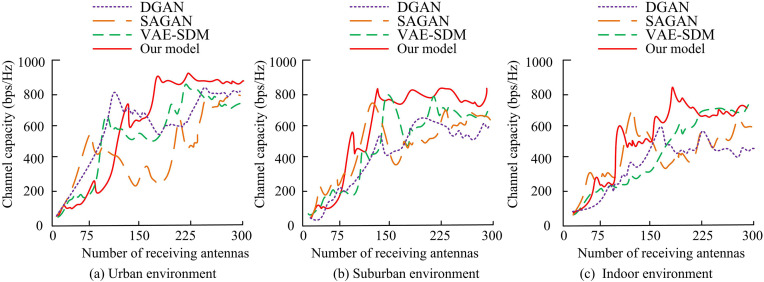
Channel capacity test results of different models in three scenarios.

The study conducted channel tests in real wireless communication environments, selecting urban and suburban environments as typical scenarios. It comprehensively collected actual channel data such as multipath fading, channel attenuation, and noise interference to evaluate the detection performance of the proposed model under real propagation conditions. By comparing channel capacity, reception rate, and error metrics across multiple scenarios, the experimental results further validated the stability and generalization ability of the model under real complex channel conditions. In particular, in urban environments, signals are affected by high-density buildings and multipath effects to simulate complex wireless propagation conditions; In suburban environments, signal propagation is relatively open and mainly affected by distance and weather changes. The indoor environment mainly considers the reflection of walls and objects, multipath fading, and signal interference between devices. The channel capacity of different detection models in three types of environments is shown in Fig 10.

**Fig 11:**
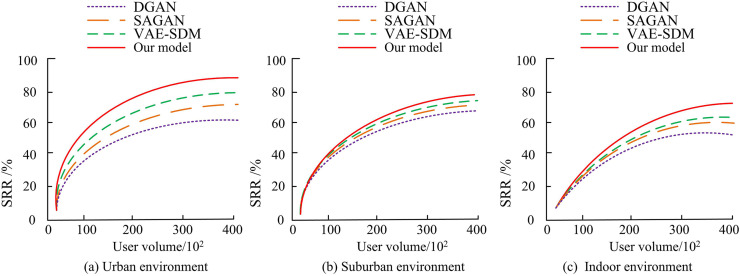
The SRR test results of different models in three scenarios.

Fig 10a–10c show channel capacity detection data in urban, suburban, and indoor environments. In urban environments, the channel capacity of the designed model continues to grow with the increase of the number of receiving antennas, reaching up to 929bps/Hz, significantly higher than DGAN’s 617bps/Hz, SAGAN’s 653bps/Hz, and VAE-SDM’s 731bps/Hz. In both suburban and indoor environments, the channel capacity of the research model reaches 852bps/Hz and 753bps/Hz with 150 and 200 receiving antennas, which are also superior to other models. The proposed model exhibits high channel capacity in various environments, especially in urban and suburban environments, where its channel capacity advantage is more pronounced. This study continues to test the SRRs of different users in three different environments, as shown in Fig 11.

**Fig 12:**
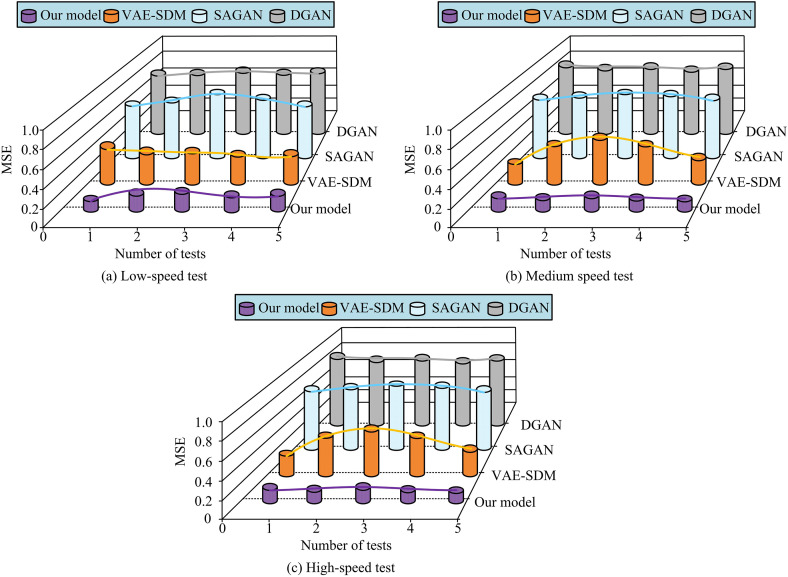
Signal detection at different moving speeds MSE test results.

In Fig 11a, in urban environments, as the number of users increases, the SRR of the research model is obviously better than other models, approaching 91.2%. The SRR of VAE-SDM and SAGAN are 62.4% and 59.8% at 100 users, while the SRR of DGAN remain below 55.4%. In the suburban environment shown in Fig 11b, the SRR of the research model is close to 87.7% at 200 users, while the SRR of other models is still relatively low. In the indoor environment shown in Fig 11c, as users rise, the SRR of all models tends to stabilize, with the research models remaining at around 78.2%, VAE-SDM at 63.8%, SAGAN at 60.3%, and DGAN performing the worst at around 54.4%. Overall, the proposed model consistently performs well in urban and suburban environments with high SRR, while in indoor environments, the SRR of all models has decreased, but the research models still maintain strong advantages. This study continues to test the signal detection of OFDM systems at low (3 km/h), medium (30 km/h), and high (120 km/h) moving speeds, as shown in Fig 12.

Fig 12a–12c show the MSE results of signal detection under low-speed (3 km/h)/medium speed (30 km/h)/high-speed (120 km/h) movement. In low-speed mobile environments, the MSE performance of the research model is optimal, consistently maintaining a low level below 0.3. The MSE of DGAN, SAGAN, and VAE-SDM is relatively high, approaching 0.7, 0.6, and 0.5. In a medium speed environment, the research model still maintains a low MSE of approximately 0.4. The errors of other models gradually increase, with DGAN and SAGAN errors of about 0.8 and 0.7, and VAE-SDM slightly lower. In high-speed mobile environments, the MSE of all models significantly increases, especially DGAN, which has an MSE close to 1, indicating poor performance at high speeds. Although the error of the research model has slightly increased, it still remains below 0.6, which is better than the comparative model. Overall, the proposed model exhibits strong robustness and low MSE at various moving speeds, especially in low-speed and medium speed environments. Finally, this study uses FOR, SER, and CEE as indicators, as shown in Table 3.

**Table 3:** Model indexes in three types of environments.

Environment	Model	FOR/%	SER/%	CEE/%
Urban	DGAN	1.54	3.88	6.42
	SAGAN	1.48	3.29	6.17
	VAE-SDM	1.72	4.02	6.63
	Our model	1.12	2.49	4.95
Suburban	DGAN	1.39	4.15	5.83
	SAGAN	1.27	3.74	5.51
	VAE-SDM	1.56	4.29	5.98
	Our model	0.92	2.99	4.28
Indoor	DGAN	1.61	3.96	6.15
	SAGAN	1.43	3.47	5.84
	VAE-SDM	1.79	4.05	6.28
	Our model	1.05	2.69	5.02

In Table 3, in urban environments, the FOR of the proposed model is 1.12%, which is lower than DGAN’s 1.54%, SAGAN’s 1.48%, and VAE-SDM’s 1.72%. SER is 2.49%, significantly lower than DGAN’s 3.88%, SAGAN’s 3.29%, and VAE-SDM’s 4.02%. CEE is 4.95%, which is also better than other models. In suburban environments, the proposed model’s FOR of 0.92%, SER of 2.99%, and CEE of 4.28% still maintain low levels, especially significantly lower than other models on FOR. In indoor environments, the FOR of the proposed model is 1.05%, SER is 2.69%, and CEE is 5.02%, all of which are better than DGAN, SAGAN, and VAE-SDM, indicating that the model can still maintain good detection performance in complex environments.

## 4. Conclusion

In response to the low signal DA of traditional OFDM systems in complex wireless channel environments, this study proposed a MIMO-OFDM model based on improved CGAN. It’s performance in various environments was evaluated through experiments using the WIMC-DS and DeepMIMO-DS datasets. In the experiment, when the weight parameter of the loss function was set to 0.7 and the regularization parameter was 0.001, the minimum MSE of the model could reach 0.2. Compared to the separate GAN and CGAN modules, after integrating CVNN and CIMSC, the BER pair of the model was 10^−5^ when the SNR was 30dB, which was relatively lower. Compared with the other three models, the lowest CEE of the research model was 1.23%. In urban environments, the model’s CEE was 1.23%, significantly lower than DGAN, SAGAN, and VAE-SDM, with an SRSR of 98.72% and DA of 96.45%. On the DeepMIMO-DS dataset, the proposed model had a CEE of 1.13%, SRSR of 99.05%, and DA of 97.25%. The ADT of 12.05ms and 11.62ms demonstrate the computational efficiency advantage of this model. In summary, the proposed model exhibits higher signal DA and stronger robustness in various environments, especially demonstrating excellent adaptability in complex environments. However, this study also has certain limitations, such as being validated only in specific wireless channel environments and having limited testing in multi-user scenarios. Future research will concentrate on the applicability of extended models in a wider range of scenarios, especially performance optimization in high-density user environments.

## Supporting information

S1 File Minimal data set definition. (DOCX)
