# Supplementary material for: Conditional generative adversarial network technology for OFDM system receiver signal detection
Source: PLoS One. 2025 Oct 14;20(10):e0334044. doi: 10.1371/journal.pone.0334044 (PMC12520388; doi:10.1371/journal.pone.0334044)
Supplement: S1 File — (DOCX) [file pone.0334044.s001.docx]

**Figure 8 Hyperparameter selection test result**

| Test | Subcarrier | 0.1 | 0.01 | 0.001 | 0.0001 | 0.2 | 0.3 | 0.4 | 0.5 | 0.6 | 0.7 | 0.8 | 0.9 |
| --- | --- | --- | --- | --- | --- | --- | --- | --- | --- | --- | --- | --- | --- |
| Loss weight | 1 | / | / | / | / | / | 0.9 |  | 0.6 |  | 0.4 |  | 0.55 |
|  | 2 | / | / | / | / | / | 0.85 |  | 0.58 |  | 0.42 |  | 0.53 |
|  | 3 | / | / | / | / | / | 0.8 |  | 0.57 |  | 0.43 |  | 0.52 |
|  | 4 | / | / | / | / | / | 0.78 |  | 0.56 |  | 0.44 |  | 0.51 |
|  | 5 | / | / | / | / | / | 0.76 |  | 0.56 |  | 0.45 |  | 0.5 |
|  | 6 | / | / | / | / | / | 0.74 |  | 0.55 |  | 0.46 |  | 0.49 |
|  | 7 | / | / | / | / | / | 0.75 |  | 0.55 |  | 0.46 |  | 0.48 |
|  | 8 | / | / | / | / | / | 0.77 |  | 0.55 |  | 0.47 |  | 0.47 |
|  | 9 | / | / | / | / | / | 0.8 |  | 0.56 |  | 0.47 |  | 0.46 |
|  | 10 | / | / | / | / | / | 0.82 |  | 0.57 |  | 0.48 |  | 0.45 |
| Regularization | 1 | 0.7 | 0.55 | 0.2 | 0.25 | / | / | / | / | / | / | / | / |
|  | 2 | 0.69 | 0.56 | 0.22 | 0.27 | / | / | / | / | / | / | / | / |
|  | 3 | 0.68 | 0.57 | 0.23 | 0.28 | / | / | / | / | / | / | / | / |
|  | 4 | 0.68 | 0.58 | 0.24 | 0.3 | / | / | / | / | / | / | / | / |
|  | 5 | 0.67 | 0.59 | 0.25 | 0.32 | / | / | / | / | / | / | / | / |
|  | 6 | 0.67 | 0.6 | 0.26 | 0.33 | / | / | / | / | / | / | / | / |
|  | 7 | 0.67 | 0.61 | 0.27 | 0.34 | / | / | / | / | / | / | / | / |
|  | 8 | 0.68 | 0.62 | 0.28 | 0.35 | / | / | / | / | / | / | / | / |
|  | 9 | 0.69 | 0.63 | 0.29 | 0.36 | / | / | / | / | / | / | / | / |
|  | 10 | 0.7 | 0.64 | 0.3 | 0.37 | / | / | / | / | / | / | / | / |
| Fading factor | 1 | / | / | / | / | 0.75 |  | 0.45 |  | 0.35 |  | 0.5 | / |
|  | 2 | / | / | / | / | 0.74 |  | 0.46 |  | 0.36 |  | 0.49 | / |
|  | 3 | / | / | / | / | 0.73 |  | 0.47 |  | 0.36 |  | 0.49 | / |
|  | 4 | / | / | / | / | 0.72 |  | 0.48 |  | 0.37 |  | 0.48 | / |
|  | 5 | / | / | / | / | 0.72 |  | 0.48 |  | 0.37 |  | 0.48 | / |
|  | 6 | / | / | / | / | 0.71 |  | 0.49 |  | 0.37 |  | 0.47 | / |
|  | 7 | / | / | / | / | 0.71 |  | 0.49 |  | 0.37 |  | 0.47 | / |
|  | 8 | / | / | / | / | 0.71 |  | 0.49 |  | 0.38 |  | 0.47 | / |
|  | 9 | / | / | / | / | 0.72 |  | 0.5 |  | 0.38 |  | 0.48 | / |
|  | 10 | / | / | / | / | 0.73 |  | 0.51 |  | 0.38 |  | 0.48 | / |
| Conv layer depth | 1 | 0.7 | 0.3 | 0.28 | 0.4 | / | / | / | / | / | / | / | / |
|  | 2 | 0.69 | 0.31 | 0.28 | 0.41 | / | / | / | / | / | / | / | / |
|  | 3 | 0.68 | 0.31 | 0.29 | 0.41 | / | / | / | / | / | / | / | / |
|  | 4 | 0.67 | 0.32 | 0.29 | 0.42 | / | / | / | / | / | / | / | / |
|  | 5 | 0.66 | 0.32 | 0.3 | 0.42 | / | / | / | / | / | / | / | / |
|  | 6 | 0.65 | 0.33 | 0.3 | 0.43 | / | / | / | / | / | / | / | / |
|  | 7 | 0.65 | 0.33 | 0.31 | 0.43 | / | / | / | / | / | / | / | / |
|  | 8 | 0.64 | 0.34 | 0.31 | 0.44 | / | / | / | / | / | / | / | / |
|  | 9 | 0.64 | 0.34 | 0.32 | 0.44 | / | / | / | / | / | / | / | / |
|  | 10 | 0.63 | 0.34 | 0.32 | 0.45 | / | / | / | / | / | / | / | / |

**Figure 9 Ablation test results**

| Dataset | SNR(dB) | GAN | CGAN | CGAN-CVNN | CGAN-CVNN-CIMSC |
| --- | --- | --- | --- | --- | --- |
| WIMC-DS | 0 | 100 | 100 | 0.018 | 0.005 |
|  | 5 | 120 | 80 | 0.008 | 0.002 |
|  | 10 | 110 | 65 | 0.007 | 0.001 |
|  | 15 | 115 | 55 | 0.005 | 0.001 |
|  | 20 | 118 | 45 | 0.004 | 0.001 |
|  | 25 | 119 | 40 | 0.004 | 0.009 |
|  | 30 | 120 | 38 | 0.003 | 0.008 |
| DeepMIMO-DS | 0 | 300 | 200 | 0.051 | 0.024 |
|  | 5 | 200 | 150 | 0.042 | 0.012 |
|  | 10 | 150 | 100 | 0.043 | 0.008 |
|  | 15 | 120 | 80 | 0.039 | 0.005 |
|  | 20 | 110 | 65 | 0.022 | 0.003 |
|  | 25 | 105 | 50 | 0.015 | 0.002 |
|  | 30 | 100 | 45 | 0.017 | 0.001 |

**Figure 10 Channel capacity test results of different models in three scenarios**

| Scenario | Antennas | DGAN | SAGAN | VAE-SDM | Our model |
| --- | --- | --- | --- | --- | --- |
| Urban | 50 | 100 | 80 | 120 | 150 |
|  | 75 | 250 | 220 | 300 | 320 |
|  | 100 | 500 | 450 | 520 | 600 |
|  | 150 | 700 | 650 | 720 | 950 |
|  | 200 | 800 | 750 | 850 | 920 |
|  | 250 | 850 | 800 | 880 | 910 |
|  | 300 | 870 | 820 | 900 | 900 |
| Suburban | 50 | 80 | 90 | 100 | 120 |
|  | 75 | 200 | 230 | 260 | 300 |
|  | 100 | 400 | 420 | 480 | 520 |
|  | 150 | 600 | 630 | 680 | 750 |
|  | 200 | 700 | 720 | 800 | 880 |
|  | 250 | 750 | 780 | 850 | 900 |
|  | 300 | 780 | 820 | 870 | 930 |
| Indoor | 50 | 60 | 70 | 90 | 100 |
|  | 75 | 150 | 180 | 200 | 220 |
|  | 100 | 300 | 320 | 350 | 400 |
|  | 150 | 450 | 480 | 500 | 600 |
|  | 200 | 550 | 600 | 650 | 720 |
|  | 250 | 600 | 650 | 700 | 780 |
|  | 300 | 650 | 700 | 750 | 800 |

**Figure 11 The acceptance rate test results of different models in three scenarios**

| Scenario | User volume (x10^2) | DGAN | SAGAN | VAE-SDM | Our model |
| --- | --- | --- | --- | --- | --- |
| Urban | 0 | 10 | 12 | 15 | 18 |
|  | 50 | 35 | 40 | 45 | 50 |
|  | 100 | 50 | 55 | 60 | 65 |
|  | 200 | 65 | 70 | 75 | 82 |
|  | 300 | 72 | 78 | 82 | 88 |
|  | 400 | 78 | 83 | 87 | 92 |
| Suburban | 0 | 8 | 10 | 12 | 15 |
|  | 50 | 30 | 35 | 38 | 45 |
|  | 100 | 45 | 50 | 55 | 60 |
|  | 200 | 60 | 65 | 70 | 78 |
|  | 300 | 68 | 74 | 78 | 85 |
|  | 400 | 73 | 79 | 84 | 90 |
| Indoor | 0 | 5 | 7 | 10 | 12 |
|  | 50 | 25 | 30 | 33 | 40 |
|  | 100 | 40 | 45 | 50 | 55 |
|  | 200 | 55 | 60 | 65 | 72 |
|  | 300 | 62 | 68 | 72 | 78 |
|  | 400 | 67 | 72 | 76 | 82 |

**Figure 12 Signal detection at different moving speeds MSE test results**

| Scenario | Test No. | Our model | VAE-SDM | SAGAN | DGAN |
| --- | --- | --- | --- | --- | --- |
| Low-speed | 1 | 0.22 | 0.55 | 0.71 | 0.98 |
|  | 2 | 0.25 | 0.55 | 0.72 | 0.95 |
|  | 3 | 0.28 | 0.57 | 0.74 | 0.98 |
|  | 4 | 0.32 | 0.58 | 0.76 | 1.04 |
|  | 5 | 0.32 | 0.66 | 0.78 | 1.02 |
| Medium-speed | 1 | 0.25 | 0.55 | 0.75 | 0.95 |
|  | 2 | 0.28 | 0.64 | 0.78 | 1.44 |
|  | 3 | 0.33 | 0.65 | 0.83 | 1.02 |
|  | 4 | 0.32 | 0.63 | 0.82 | 1.05 |
|  | 5 | 0.35 | 0.62 | 0.85 | 1.07 |
| High-speed | 1 | 0.37 | 0.67 | 0.81 | 1.86 |
|  | 2 | 0.32 | 0.62 | 0.82 | 1.02 |
|  | 3 | 0.35 | 0.65 | 0.85 | 1.05 |
|  | 4 | 0.37 | 0.68 | 0.87 | 1.08 |
|  | 5 | 0.38 | 0.79 | 0.94 | 1.19 |
